# Performance and milk fatty acid profile of beef cows with a different energy status with short nutrient restriction and refeeding

**DOI:** 10.1093/jas/skad053

**Published:** 2023-02-16

**Authors:** Karina G Orquera-Arguero, Mireia Blanco, Juan R Bertolín, Javier Ferrer, Isabel Casasús

**Affiliations:** Centro de Investigación y Tecnología Agroalimentaria de Aragón (CITA), Avda. Montañana 930, 50059, Zaragoza, Spain; Instituto Agroalimentario de Aragón – IA2 (CITA-Universidad de Zaragoza), Zaragoza, Spain; Centro de Investigación y Tecnología Agroalimentaria de Aragón (CITA), Avda. Montañana 930, 50059, Zaragoza, Spain; Instituto Agroalimentario de Aragón – IA2 (CITA-Universidad de Zaragoza), Zaragoza, Spain; Centro de Investigación y Tecnología Agroalimentaria de Aragón (CITA), Avda. Montañana 930, 50059, Zaragoza, Spain; Instituto Agroalimentario de Aragón – IA2 (CITA-Universidad de Zaragoza), Zaragoza, Spain; Centro de Investigación y Tecnología Agroalimentaria de Aragón (CITA), Avda. Montañana 930, 50059, Zaragoza, Spain; Centro de Investigación y Tecnología Agroalimentaria de Aragón (CITA), Avda. Montañana 930, 50059, Zaragoza, Spain; Instituto Agroalimentario de Aragón – IA2 (CITA-Universidad de Zaragoza), Zaragoza, Spain

**Keywords:** beef cows, induced feed restriction, metabolites, milk fatty acid profile, refeeding

## Abstract

Our study objective was to determine the effect of a short feed restriction (4 d) and subsequent refeeding (4 d) on the performance and metabolism of beef cows with a different nutritional status by particularly focusing on their milk fatty acid (**FA**) profile, to consider its potential use as biomarker of metabolic status. Thirty-two Parda de Montaña multiparous lactating beef cows were individually fed a diet based on the average cow’s net energy (**NE**) and metabolizable protein requirements. At 58 d in milk (**DIM**, day 0), cows underwent a 4 d feed restriction (55% requirements, restriction period). Before and after the restriction, diets met 100% of their requirements (basal and refeeding periods). Cow performance, milk yield and composition, and plasma metabolites, were determined on day −2, 1, 3, 5, 6, and 8. Cows were classified into two status clusters according to their pre-challenge performance and energy balance (**EB**) (Balanced vs. Imbalanced). All traits were statistically analyzed considering the fixed effect of status cluster and feeding period or day, with cow as a random effect. Imbalanced cows were heavier and had a more negative EB (*P* < 0.001), but similar milk yield, milk composition, and circulating metabolites (except for greater urea) than Balanced cows (*P* > 0.10). Milk contents of C18:1 cis-9, monounsaturated FA (**MUFA**), and mobilization FA were greater (*P* < 0.05), whereas saturated FA (**SFA**) and de novo FA were lesser in Imbalanced than Balanced cows (*P* < 0.05). Restriction decreased body weight (**BW**), milk yield, and milk protein compared to the basal period, but increased milk urea and plasma nonesterified fatty acids (**NEFA**) (*P* < 0.001). Milk contents of SFA, de novo, and mixed FA decreased immediately during the restriction, while MUFA, polyunsaturated FA and mobilization FA increased (*P* < 0.001). Basal milk FA contents were recovered on day 2 of refeeding, and all their changes strongly correlated with differences in EB and NEFA (*P* < 0.05). The general lack of interactions between status clusters and feeding periods implied that the response mechanisms to diet changes did not differ between cows with a different pre-challenge nutritional status.

## Teaser

The pattern of reaction to and recovery from a short feed restriction during lactation was similar among beef cows in different nutritional status.

Milk fatty acid composition in beef cattle is an indicator of metabolic status and body fat mobilization during feed shortage.

## Introduction

Wide seasonal variations in the availability and quality of feeding resources in extensive ruminant systems imply that animals are often subjected to underfeeding-refeeding cycles (Bocquier and González-García, 2010). When undernutrition occurs in lactating cows, both homeostatic and homeorhetic controls bring about adaptations to help to maintain balance and to supply nutrients to the mammary gland (Bauman and Currie, 1980) to support the high metabolic priority of milk production. Strategies to cope with the physiological imbalance caused by feed restriction depend, among other factors, on: restriction duration and its severity (Leduc et al., 2021); lactation stage (Orquera-Arguero et al., 2022); individual variability (Gross et al., 2011a; Bjerre-Harpøth et al., 2012). In beef cows, the impacts of restriction and refeeding on cow metabolism have been well assessed in the long term (Fiems et al., 2015), and only recently with short-term restrictions (De La Torre et al., 2022; Orquera-Arguero et al., 2022). Furthermore, ad libitum or individual feeding strategies are commonly used in dairy cattle, where individual concentrate allocation based on milk yield can improve the energy balance (**EB**) and cow performance (Lawrence et al., 2016), while other studies report no milk yield differences (Henriksen et al., 2019). On extensive beef cow farms, feeding management is often simplified by adopting a flat-rate regime (Manninen et al., 2004), which involves all cows receiving the same diet irrespectively of their individual requirements. This common feeding can cause disruptive situations under an eventual restriction in nutrient intake, with the most sensitive individuals, those with greater requirements, being the most affected (Bocquier and González-García, 2010). Clustering analyses have been used to group dairy cows according to their performance, plasma metabolites, hormones, and milk traits to identify animals with different strategies to face metabolic challenges (De Koster et al., 2019; Xu et al., 2019; Orquera-Arguero et al., 2022), which could facilitate herd management decisions.

Major changes occur in adipose tissue in response to a negative EB, which results in the mobilization of body reserves and an increase in circulating nonesterified fatty acids (**NEFA**) and ketones to provide energy and precursors for milk synthesis (Baumgard et al., 2017). Plasma concentrations of these and other metabolites, such as malondialdehyde (**MDA**), associated with oxidative status (Castillo et al., 2006) or urea as an indicator of protein metabolism (Bittante, 2022), have been used as biomarkers of cow metabolic load. In the last few years, milk composition traits have been examined as non-invasive indicators of dairy cows’ nutritional status (Gross and Bruckmaier, 2019; Billa et al., 2020) because they can be cost-efficiently and routinely measured from test-day milk samples (Mäntysaari et al., 2019). Of them, milk fatty acid (**FA**) contents are promising indicators of energy status in dairy cows (Khiaosa-ard et al., 2020) given that FA C4:0 to C14:0 are synthesized *de novo* in the mammary gland, whereas those longer than C18:0 and around 50% of C16:0 originate from diet and lipid mobilization (Chilliard et al., 2000; Palmquist, 2009). In fact C16:0, C18:0, and 18:1 cis-9 are the most abundant FA in plasma and body fat stores (Hostens et al., 2012), and their concentrations and ratios are closely related to the EB in dairy cows (Dórea et al., 2017), but no information on this is available in beef cows. We hypothesized that the response to restriction and refeeding would be driven by each cow’s weight, milk yield, and nutritional status before the challenge. Therefore, the main objectives of this study were to: 1) evaluate the effects of a negative EB induced by a short feed restriction on the performance, metabolites, and milk FA profile in two groups of beef cows classified according to their previous performance; 2) confirm the potential use of milk FA composition as a biomarker of metabolic status in beef cows.

## Materials and Methods

The Animal Ethics Committee of the Research Centre approved the experimental procedures (protocol no. CEEA-03-2018-01), which followed the guidelines of EU Directive 2010/63 on the protection of animals used for experimental and other specific purposes (EU, 2010). The experiment was conducted in the Pyrenees Mountain area at the CITA La Garcipollera Research Station (Spain, 42°37ʹ N, 0°30ʹ W, 945 m a.s.l.).

### Animal management, diets, and experimental design

The study was conducted with 32 multiparous Parda de Montaña beef cows (at calving: body weight [**BW**]: 626 ± 47.7 kg; body condition score [**BCS**, on a 5-point scale]: 2.8 ± 0.22; age: 7.5 ± 2.91 yr). One cow was removed from the study due to physical injury. After calving, cows were randomly allocated in pens (eight cows/pen, 10 × 20 m) equipped with individual feeders for forage (200-l fiberglass boxes in front of self-locking feeding places) and automatic feeding stations (ALPRO Herd Management 7.0, DeLaval) for concentrate. Calves were penned in straw-bedded cubicles adjacent to their dams. They were allowed to suckle their dams twice daily for 30 min at 06:00 h and 14:00 h.

Cows were fed a flat-rate regime during lactation. They all received the same amount of feed. Diets were calculated by considering the net energy (**NE**) and metabolizable protein requirements for the maintenance and lactation of a standard cow (615 kg BW; milk yield: 8.5 kg/d) using INRA equations (INRA, 2007). From calving to the start of the experiment 2 mo later, cows were fed a formulated diet to meet 100% standard cow energy requirements (Table 1).

**Table 1.** Chemical composition, fatty acids (FA) composition and nutrition value (mean ± SD) of the feedstuffs offered to beef cows

Parameter	Hay	Concentrate
Chemical composition		
DM^3^, g/kg	922 ± 11.7	906 ± 4.0
Ash, g/kg DM	86.4 ± 24.4	68.3 ± 1.4
Crude protein, g/kg DM	109 ± 18.3	167 ± 4.7
Neutral detergent fiber, g/kg DM	570 ± 52.4	256 ± 23.2
Acid detergent fiber, g/kg DM	324 ± 32.9	114 ± 11.1
Lignin, g/kg DM	35.2 ± 12.8	29.4 ± 8.8
FA composition		
C16:0, g/100 g ID FAME^1^	32.2 ± 2.37	19.2 ± 0.60
C18:0, g/100 g ID FAME^1^	14.1 ± 2.02	5.3 ± 0.02
C18:1 cis-9, g/100 g ID FAME^1^	4.5 ± 1.15	23.6 ± 0.32
C18:2 n-6, g/100 g ID FAME^1^	15.7 ± 3.30	44.4 ± 1.78
C18:3 n-3, g/100 g ID FAME^1^	26.6 ± 10.17	1.8 ± 0.31
Total, mg ID FAME^1^/g DM	18.5 ± 2.99	65.7 ± 2.15
Nutritive value		
Net energy, MJ/kg DM	5.5 ± 0.15	7.3 ± 0.41
Metabolizable protein, g PDI^2^/kg DM	81 ± 17.9	123 ± 2.4

^1^Identified fatty acid methyl esters.

^2^True protein digestible in the small intestine.

^3^DM, dry matter.

The experiment was conducted at the end of the second lactation month and involved three consecutive periods, where day 0 was taken as the first day of restriction (days in milk [**DIM**]: 58 ± 6.3). Cows were first fed a diet that met 100% of their energy and metabolizable protein requirements (day −2 to −1, basal period), then 55% of those requirements for 4 d (day 0 to 3, restriction period) and, finally, 100% again on the following 4 d (day 4 to 7, refeeding period). Diets consisted of 8.0 kg hay and 3.0 kg of concentrate (as-fed basis) during the basal and refeeding periods, and 7.0 kg hay during the restriction period. ­Animals had free access to water and mineral blocks throughout the experiment.

### Measurements

Samples of feedstuffs were collected daily (day −2 to 8) and lyophilized in a Genesis Freeze Dryer 25 (Hucoa Erlöss, SA/Thermo Fisher Scientific) to determine their chemical composition and FA profile. Hay was offered daily at 08:00 h as a single meal in individual troughs, where cows were tied up until they finished their ration, during approximately 2 h. ALPRO feeding stations were programmed to offer 3 kg of concentrate daily (as-fed basis) to all the cows during the basal and refeeding periods. Individual concentrate intake was recorded daily.

The BCS was recorded upon calving, 30 DIM, and on experimental period day −2 and 8. It was determined by a trained person on a 1–5 scale, based on estimating the fat covering ribs, loin, and tailhead (Lowman et al., 1976). Cows were weighed on an electronic scale upon calving and then at 07:00 h on 30 and 31 DIM and on experiment day −2, 1, 3, 5, 6, and 8. Milk yield was estimated on the same days by the weight-suckle-weight technique (Le Neindre and Dubroeucq, 1973). Calves were weighed before and after the two daily 30-min periods in which they had access to suckle their dams. The daily milk yield was estimated as the sum of the milk consumed by the calf in these two suckling periods. Milk samples were manually taken from each dam after the morning suckling. Five minutes before the manual extraction, all cows received an intramuscular injection of oxytocin (40 UI, Facilpart, Laboratorios Syva, León, Spain) to accelerate the letdown of the residual milk. A 100-mL sample was collected to determine milk composition, added with sodium azide (PanReac) as a preservative and refrigerated at 4 °C until the analysis. To determine FA composition, a second 40-mL sample was collected, lyophilized, and stored at −20 °C until analyzed.

Cows were bled on the same experiment days described above to assess their metabolic profile. Blood samples were collected from the coccygeal vein at 07:00 h after suckling and before offering hay. Heparinized tubes (BD Vacutainer Becton-Dickenson and Company) were used for the β-hydroxybutyrate (**BHB**) and MDA determinations, and the tubes that contained K2 EDTA (BD Vacutainer Becton-Dickenson and Company) were used to analyze glucose, NEFA, and urea concentrations. Immediately after collection, blood samples were centrifuged at 3,500 rpm for 20 min at 4 °C. Plasma was collected and frozen at −20 °C until further analyses.

### Analyses

#### Feedstuffs and milk

The chemical composition of feedstuffs was analyzed in duplicate as described in Orquera-Arguero et al. (2022). Briefly, dry matter (**DM**) and ash content were determined according to AOAC methods (AOAC, 2000). Nitrogen content was determined following the Dumas Procedure (index no. 968.06) with a nitrogen analyzer (Model NA 2100, CE Instruments, Thermoquest SA., Barcelona, Spain). Neutral detergent fiber, acid detergent fiber, and acid detergent lignin contents were analyzed following the sequential procedure of Van Soest et al. (1991) with an Ankom 200/220 fiber analyzer (Ankom Technology Corporation, Fairport, NY, USA). In milk samples, fat, protein, and urea contents were analyzed by an infrared scan (Milkoscan 7 RM, Foss Electric Ltd., Hillerød, Denmark). The FA of the freeze-dried feedstuffs were extracted and methylated as proposed by Sukhija and Palmquist (1988). The fatty acid methyl esters (**FAME**) of the freeze-dried milk samples were obtained as described by Kramer et al. (1997). Determination was done by gas chromatography with a flame ionization detector and Bruker Scion 436-GC (Bruker, Billerica, USA) equipped with a CP-8400 Autosampler (Bruker), a cyanopropyl capillary column SP-2560 (100 m × 0.25 mm ID × 0.20 µm thickness for feedstuffs and 200 m × 0.25 mm ID × 0.20 µm thickness for milk) (Sigma-Aldrich, Sant Louis, USA) and the Compass CDS software. FAME was ID using the GLC-532, GLC-401, GLC-643, GLC-642, GLC-463 C18:1 t11, C19:0, C23:0 (Nu-Chek-Prep Inc.), mixture BR1, mixture BR4 (Larodan Research Grade Lipids) standard references, and the relative retention times observed in the bibliography (Kramer et al., 1997; Shingfield et al., 2003; De La Fuente et al., 2015). Fatty acid quantification was performed as described in UNE-EN ISO 12966-4:^2015^ and expressed as a percentage of the total amount of identified FAME. The chemical composition and FA profile of the feedstuffs are presented in Table 1.

#### Blood metabolites

Glucose (enzymatic-colorimetric method, sensitivity: 0.06 mmol/L) and urea (kinetic method, sensitivity: 0.056 mmol/L) concentrations were determined in plasma with an automatic analyzer (Gernon, RAL S.A, Barcelona, Spain). The mean intra- and interassay CV were 1.5% and 1.9% for glucose and 3.2% and 4.8% for urea, respectively. Plasma BHB (kinetic enzymatic method, sensitivity: 0.100 mmol/L) and NEFA (colorimetric method, sensitivity: 0.072 mmol/L) were determined using Randox kits (Randox Laboratories Ltd., Country Antrim, UK). The mean intra- and interassay CV were respectively 3.3% and 3.7% for NEFA and 6.2% in both cases for BHB. Oxidative status was determined using MDA as a biomarker of lipid peroxidation. This indicator was determined by liquid chromatography using an Acquity UPLC H-Class liquid chromatograph (Waters, Milford, MA, USA) equipped with a silica-based bonded phase column (Acquity UPLC HSS PFP, 100 mm × 2.1 mm × 1.8 μm, Waters), an absorbance detector (Acquity UPLC Photodiode Array PDA eλ detector, Waters) and a fluorescence detector (2475 Multi λ Fluorescence Detector, Waters). The quantification of MDA was done by fluorescence detection at ʎ_excitation_ = 530 nm and ʎ_emission_ = 550 nm following the chromatographic conditions described in Bertolín et al. (2019). The mean intra- and interassay CV were 4.6% and 7.3%, respectively.

### Calculations

The chemical composition of feedstuffs was employed to calculate their NE content using INRA equations (INRA, 2007). Individual EB was estimated by calculating the difference between inputs (NE intake) and outputs (NE for maintenance and NE for lactation) (INRA, 2007). Net energy intake was estimated from the individual intake and energy contents of feedstuffs. Net energy for maintenance was calculated from the individual metabolic weight. Net energy for production was obtained using the milk yield, fat, and protein contents in milk.

In milk, FA were grouped according to their degree of saturation as saturated fatty acid (**SFA**), monounsaturated fatty acid (**MUFA**), and polyunsaturated fatty acid (**PUFA**) ­according to their origin from de novo synthesis (C4:0–C15:1), of mixed origin (C16:0–C16:1), and from mobilization (≥C17:0) (Palmquist, 2009). The C18:1 cis-9 to C15:0 FA ratio was calculated to assess its relation with the EB and metabolic profile.

### Statistical analyses

All the data were analyzed using the SAS statistical package v 9.4 (SAS Institute, Inc., Cary, NC, USA). Cows were assigned to clusters according to their resemblance in terms of Euclidean distance calculated using data from BW and BCS at calving and BW, BCS, milk yield, and EB at 30 and 31 DIM. A non-hierarchical clustering was performed using the k-means method (FASTCLUS procedure). The selection of the optimum number of clusters was based on cubic conglomerating criteria. Two clusters (hereafter referred to as status clusters) were obtained, namely Balanced and Imbalanced. An analysis of variance was performed on the classifying variables using a general linear model (GLM procedure) and taking the cluster as a fixed effect.

Cows’ metabolic and production data were studied in two sets of analyses, which considered different time effects during the experiment: feeding period (basal, restriction, refeeding) and day (day −2 to 8). In both cases, mixed models for repeated measures (MIXED procedure) were used by considering the status cluster (Balanced and Imbalanced), time (feeding period or day), their interaction as fixed effects and cow as the random effect. The model used was Y_ijk_ = μ + S_j_ + T_k_ + S_j_ x T_k_ + C_i_ + e_ijk_, where Y_ijk_ was the dependent variable at each time point for the ith cow; μ, the overall mean; S_j_, the effect of the status cluster; T_k_, the effect of time (either feeding period or day); C_i_, the random effect of cow i and e_ijk_ was the experimental error. Degrees of freedom were adjusted with the Kenward–Roger correction to take into account missing values. The variance components structure was selected on the basis of the lowest Akaike and Bayesian information criteria. Least square means and associated standard errors were obtained, and multiple comparisons were adjusted with Tukey correction. Pearson’s relations (*r*) between variables were obtained and presented on heatmaps for cow performance, plasma metabolites, and milk FA composition variables using the CORRPLOT package of R (R Core Team, 2021). The data set used for the correlation analyses corresponds to all traits and samples collected per cow at day −2, 1, 3, 5, 6, and 8 of experiment (*n* = 186 values per trait). The *P*-value for significance was set at *P <* 0.05 and trends were discussed when 0.05 ≤ *P <* 0.10.

## Results

The results of the status cluster and feeding period effects appear in the tables. The results of the status cluster and day effects are plotted in the figures. The clustering analysis resulted in two cow clusters, which differed in terms of their pre-experimental BW, milk yield, and EB (Table 2). Cows in the first cluster were classified as Balanced and those in the second cluster as Imbalanced. Balanced cows were lighter, had a lower milk yield and a less negative EB than Imbalanced cows in the second cluster (*P* ≤ 0.03).

**Table 2.** Initial cow characteristics (30–31 d in milk) according to the status cluster^1^

Item	Balanced	Imbalanced	SEM	*P*-value
*n*	15	16	-	-
Body weight, kg	563	633	4.12	<0.001
Body condition score (scale 1 to 5)	2.8	2.9	0.04	0.18
Milk yield, kg/d	7.5	8.6	0.17	0.03
Energy balance, MJ NE^2^/d	−3.5	−10.0	0.77	<0.001

^1^Cows clustered according to the analysis based on pre-challenge cow traits and energy status.

^2^Net energy.

### Cow performance

**Figure 1.**
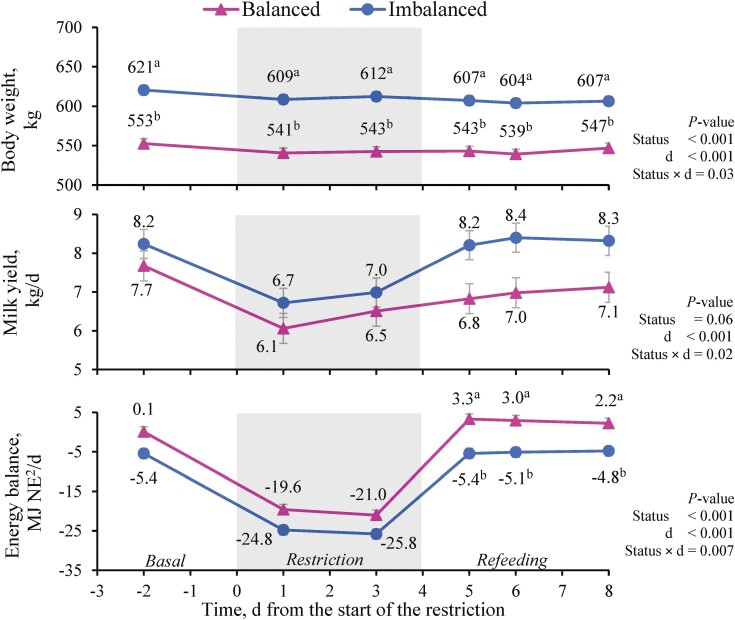
Effect of the status cluster^1^ and day (d) on beef cows’ body weight, milk yield, and energy balance. The gray area represents the 4 d feed restriction at 55% of cows’ energy and metabolizable protein requirements. Vertical bars indicate the standard error. ^1^According to the clustering analysis based on pre-challenge cow traits and energy status. ^2^ Net energy. ^a, b^ Within a day, different superscripts indicate differences between status clusters (*P* < 0.05).

Dry matter intake (**DMI**) was only affected by feeding period (*P <* 0.001; Table 3). According to the experimental design, DMI was lower during the restriction than during the basal and refeeding periods (*P <* 0.001), and so were energy intakes (59.8, 34.9, and 59.8 MJ NE/d during the basal, restriction, and refeeding periods, respectively, *P <* 0.001) and metabolizable protein intakes (859, 471, and 859 g/d, respectively; *P <* 0.001). The BCS was affected by the status cluster (2.65 and 2.81 in Balanced and Imbalanced cows, respectively, *P <* 0.001), and tended to decrease between day −2 and day 8 (2.75 and 2.71, respectively, *P =* 0.08). Cow BW was affected by the interaction between status cluster and feeding period (Table 3) because restriction decreased BW in both groups (*P* < 0.001), but during refeeding BW decreased even more in Imbalanced cows (*P* = 0.03), whereas it was maintained in Balanced cows (*P* ≥ 0.23). In any case, Balanced cows were lighter than their Imbalanced counterparts throughout the experiment (*P* < 0.001). Regarding daily changes, BW of Imbalanced cows lowered from the start (day −2) to the end of the experiment (day 8) (*P* < 0.05), while that of Balanced cows decreased until day 6 (*P* < 0.01), but then regained basal values on day 8 (Figure 1).

**Table 3.** Effect of the status cluster^1^ and FP^2^ on beef cows’ performance

	Status cluster			*P*-value		
Item	Balanced	Imbalanced	RSD^3^	Status	FP	Status × FP
Dry matter intake, kg/d			0.16	0.98	<0.001	0.51
Basal	10.0^a^	10.1^a^				
Restriction	6.4^b^	6.5^b^				
Refeeding	10.1^a^	10.0^a^				
Body weight, kg			6.55	<0.001	<0.001	0.01
Basal	553^a, y^	621^a, x^				
Restriction	542^b, y^	611^b, x^				
Refeeding	543^b, y^	606^c, x^				
Milk yield, kg/d			0.70	0.10	<0.001	0.001
Basal	7.7^a^	8.2^a^				
Restriction	6.3^c^	6.9^b^				
Refeeding	7.0^b^	8.3^a^				
EB^4^, MJ NE^5^/d			2.46	<0.001	<0.001	<0.001
Basal	0.1^b, x^	−5.4^a, y^				
Restriction	−20.3^c, x^	−25.3^b, y^				
Refeeding	2.8^a, x^	−5.1^a, y^				

^1^According to the clustering analysis based on pre-challenge cow traits and energy status.

^2^FP, feeding period.

^3^Residual standard deviation.

^4^Energy balance.

^5^Net energy.

^a,b,c^Different superscripts indicate differences between feeding periods (*P* < 0.05).

^x,y^Different superscripts indicate differences between status clusters (*P* < 0.05).

**Figure 2.**
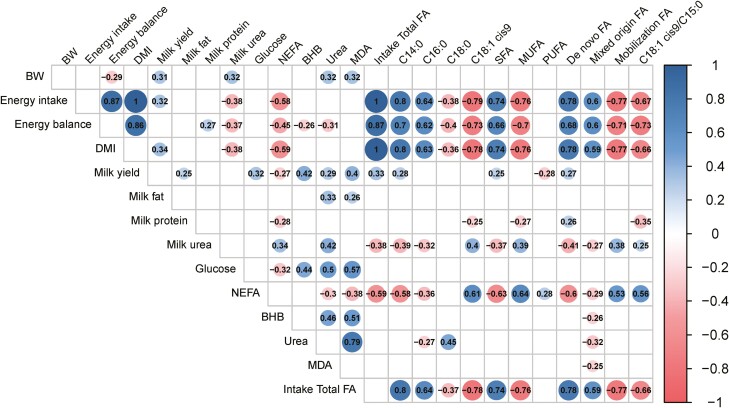
Significant Pearson’s correlations (*P* < 0.05) among beef cow performance, metabolic profile variables and milk fatty acids (FA) composition. BHB, β- hydroxybutyrate; BW, body weight; DMI, dry matter intake; MDA, malondialdehyde; MUFA, monounsaturated FA; NEFA, nonesterified fatty acids; PUFA, polyunsaturated FA; SFA, saturated FA; de novo FA (C4:0 - C15:1), mixed origin FA (C16:0 - C16:1), and mobilization FA (≥ C17:0).

Milk yield was affected by the status cluster-feeding period interaction (*P* < 0.001, Table 3). Milk yield lowered similarly during the restriction in both status clusters (−18% and −17% for Balanced and Imbalanced cows, respectively). During refeeding, it increased again to the basal values for Imbalanced cows but did not fully recover for Balanced cows (−9%). Milk yield loss due to the restriction varied between −3% and −37% among cows. On average, Imbalanced cows had a numerically, but nonsignificantly greater milk yield (7.0 vs. 7.8 kg/d in Balanced vs. Imbalanced cows, respectively, *P =* 0.10). In fact, when analyzed by day Imbalanced cows showed faster milk yield regain during the refeeding period (Figure 1). Cow EB was affected by the status cluster and feeding period interaction (*P* < 0.001) because the difference between Balanced and Imbalanced cows was greater during the refeeding period than during the basal and restriction periods (Table 3). In both groups, EB was more negative during the restriction period than in the other periods (*P* < 0.001). This was confirmed when analyzed by day, where the differences between status clusters were only significant on day 5, 6, and 8 during the refeeding period (Figure 1). Milk fat content only tended to be affected by the status cluster, with a lower content in Balanced than in Imbalanced cows (*P =* 0.09; Table 4). Milk protein and milk urea contents were affected only by feeding period (*P <* 0.001; Table 4). Milk protein content was lesser and milk urea content was greater during the restriction compared to the other periods (*P <* 0.001), which was corroborated by the negative correlation between milk urea and EB (Figure 2).

**Table 4.** Effect of the status cluster^1^ and FP^2^ on beef cows’ milk composition

	Status cluster		FP				*P*-value^4^	
Item	Balanced	Imbalanced	Basal	Restriction	Refeeding	RSD^3^	Status	FP
Fat, g/100 g	4.28	4.77	4.58	4.57	4.41	0.80	0.09	0.37
Protein, g/100 g	2.91	2.91	2.93^a^	2.85^b^	2.95^a^	0.01	0.94	<0.001
Urea, mg/dL	22.8	24.5	22.7^b^	25.5^a^	22.8^b^	2.45	0.29	<0.001

^1^According to the clustering analysis based on pre-challenge cow traits and energy status.

^2^FP, feeding period.

^3^Residual standard deviation.

^4^The interaction was never significant (*P* = 0.31–0.94).

^a,b^ Different superscripts indicate differences among feeding periods (*P* < 0.05).

### Blood metabolites

**Figure 3.**
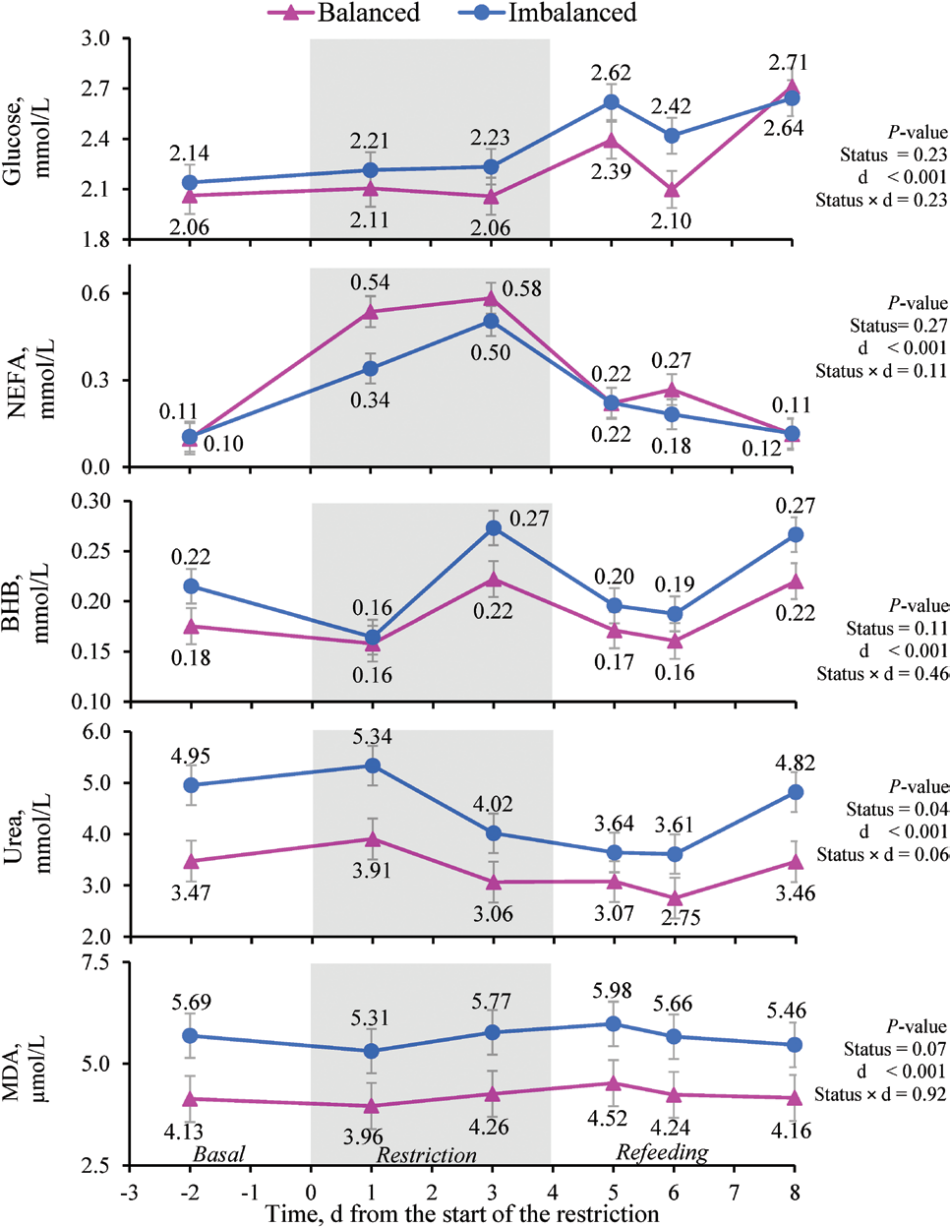
Effect of the status cluster^1^ and the day (d) on the plasma metabolites^2^ of the beef cows. The gray area represents the 4 d feed restriction at 55% of cows’ energy and metabolizable protein requirements. Vertical bars indicate the standard error. ^1^According to the clustering analysis based on pre-challenge cow traits and energy status. ^2^NEFA: nonesterified fatty acids; BHB: β- hydroxybutyrate (BHB); MDA: malondialdehyde.

Plasma glucose concentration was affected only by feeding period (*P <* 0.001; Table 5). Glucose concentrations were similar during the basal and restriction periods, but rose during the refeeding period (*P <* 0.001). Plasma NEFA concentration was affected by feeding period (*P <* 0.001, Table 5), and increased during the restriction before decreasing during the refeeding period. When NEFA concentration was analyzed by day, an immediate response to diet changes was observed, with a rise after only 1 d on the restricted diet (day 1) and the basal values recovered after 1 d of refeeding (day 5) (Figure 3). Daily NEFA concentration in plasma correlated negatively with energy intake and EB (*P <* 0.001; Figure 2). Plasma BHB concentration was not affected by either the status cluster or the feeding period (Table 5). However, when analyzed by day, minor fluctuations in BHB concentrations occurred (Figure 3). Daily plasma BHB concentration weakly, but positively, correlated with both milk yield and glucose plasma concentration (*P <* 0.001; Figure 2).

**Table 5.** Effect of the status cluster^1^ and FP^2^ on beef cows’ plasma metabolite concentrations.

	Status cluster		FP				*P*-value^4^	
Item	Balanced	Imbalance	Basal	Restriction	Refeeding	RSD^3^	Status	FP
Glucose, mmol/L	2.18	2.31	2.10^b^	2.15^b^	2.48^a^	0.35	0.28	<0.001
NEFA^5^, mmol/L	0.29	0.23	0.10^c^	0.49^a^	0.19^b^	0.17	0.33	<0.001
BHB^6^, mmol/L	0.18	0.22	0.20	0.20	0.20	0.06	0.10	0.78
Urea, mmol/L	3.35	4.55	4.21^a^	4.08^a^	3.56^b^	0.84	0.03	<0.001
MDA^7^, µmol/L	4.18	5.64	4.91	4.83	5.00	0.51	0.07	0.10

^1^According to the clustering analysis based on pre-challenge cow traits and energy status.

^2^FP, feeding period.

^3^ Residual standard deviation.

^4^ The interaction was never significant (*P* = 0.08–0.92).

^5^ Nonesterified fatty acids.

^6^ β- hydroxybutyrate.

^7^ Malondialdehyde.

^a,b,c^ Different superscripts indicate differences between feeding periods (*P* < 0.05).

Plasma urea concentrations were affected by both the status cluster (*P =* 0.03), with lesser values in Balanced than in Imbalanced cows, and the feeding period (*P <* 0.001; Table 5), with lesser concentrations during refeeding than the other periods. When plasma urea was analyzed daily (Figure 3), it decreased from day 1 of the restriction to day 6 of refeeding, and then increased and reached the basal values by the end of the experiment (day 8). Plasma urea concentration positively correlated with milk urea and plasma glucose and BHB concentrations (*P <* 0.001; Figure 2). Plasma MDA concentration tended to be affected by status cluster (*P =* 0.07; Table 5), and Balanced cows tended to have lesser concentrations than Imbalanced cows. Despite no clear differences being observed for feeding period, an increase in plasma MDA was observed by day 3 of the restriction as compared to previous basal values (*P* < 0.05) when analyzed by day (see Figure 3) and up to the start of the refeeding period (day 5 and 6). Basal values had recovered by the end of refeeding (day 8). Plasma MDA concentration positively correlated with glucose, BHB, and urea plasma concentrations (*P <* 0.001; Figure 2).

### Diet FA intake and milk FA content

**Figure 4.**
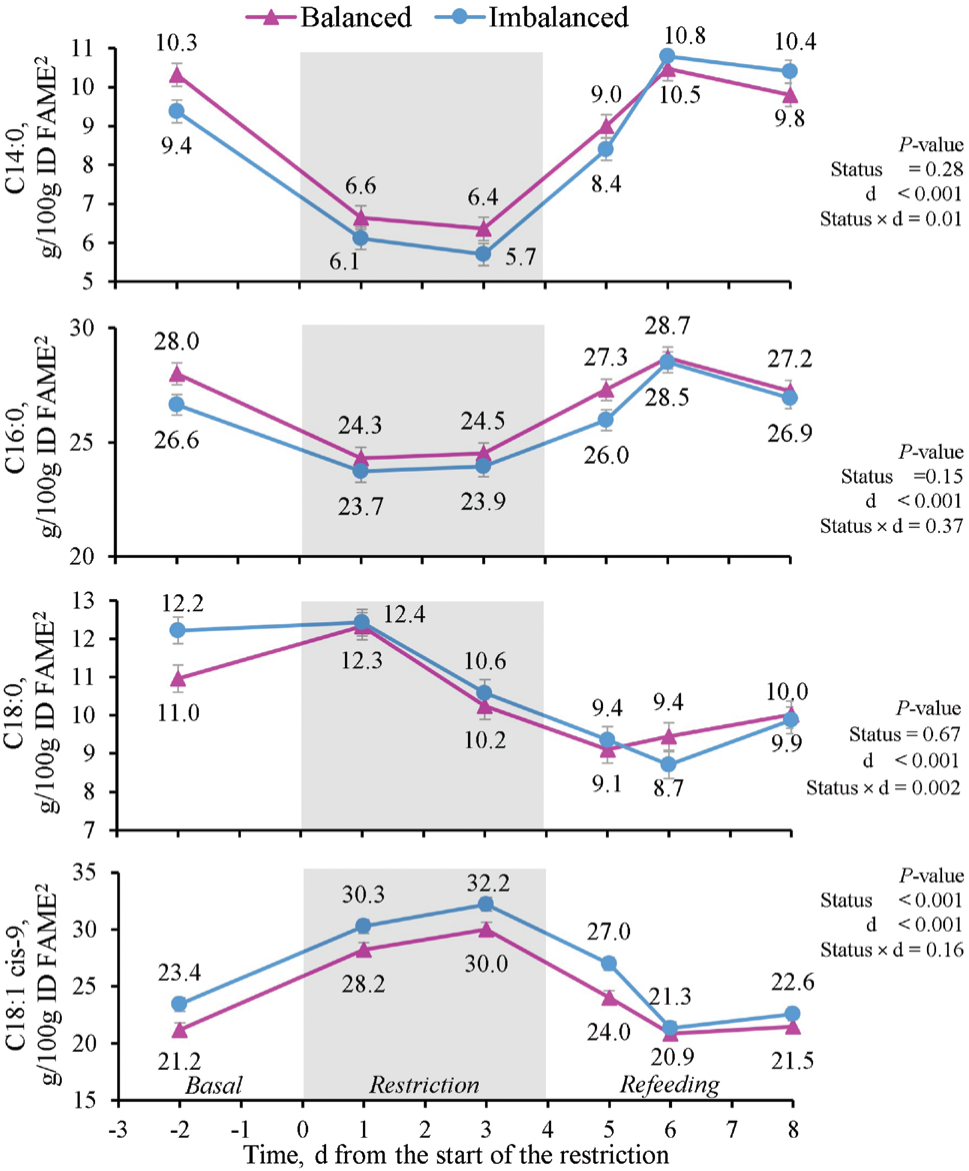
Effect of the status cluster^1^ and day (d) on beef cows’ milk concentrations of individual milk fatty acids: C14:0, C16:0, C18:0, and C18:1 cis-9. The gray area represents the 4 d feed restriction at 55% of cows’ energy and metabolizable protein requirements. Vertical bars indicate the standard error. ^1^ according to the clustering analysis based on pre-challenge cow traits and energy status. ^2^ identified fatty acid methyl esters.

Diet FA intake were affected only by feeding period (*P* < 0.001), decreased during the restriction and increased to the basal intakes during refeeding (Table 6). Regarding the individual FA in milk, the status cluster tended to affect C16:0 (*P =* 0.09) and C18:1 cis-9 (*P =* 0.002), with greater concentrations in Imbalanced than in Balanced cows. All the major milk FA were affected by feeding period (*P* < 0.001). Restriction lowered the milk contents of C14:0 and C16:0 and increased those of C18:1 cis-9. During refeeding, C14:0 and C16:0 increased, while C18:0 and C18:1 cis-9 decreased. The time effect was confirmed when analyzing C14:0 and C16:0 on a daily basis. Feed restriction elicited an immediate response with nadir values on day 1 and 3, and then increased during refeeding. With C14:0, a status cluster and day interaction (*P =* 0.01) took place because of the slightly different recovery pattern noted during refeeding (Figure 4). The C18:1 cis-9 content increased steadily on d 1 and 3 of the restriction, and then decreased on the first day of refeeding (Figure 4). Milk contents of C14:0 and C16:0 positively correlated, whereas C18:1 cis-9 correlated negatively with EB (*P <* 0.001; Figure 2). Milk C14:0 correlated negatively and C18:1 cis-9 positively with NEFA plasma content (*P <* 0.001, Figure 2).

**Table 6.** Effect of the status cluster^1^ and FP^2^ on beef cows’ dietary intake of FA^3^ and on the major FA in milk, FA according to their saturation and origin, and the C18:1 cis-9 to C15:0 ratio

	Status cluster		FP				*P*-value^5^	
Item	Balanced	Imbalanced	Basal	Restriction	Refeeding	RSD^4^	Status	FP
Intake of dietary FA, g/d								
C16:0	64.3	64.1	77.2^a^	38.1^b^	77.2^a^	2.01	0.55	<0.001
C18:0	24.4	24.4	28.3^a^	16.6^b^	28.3^a^	0.56	0.74	<0.001
C18:1 cis-9	33.5	33.1	47.4^a^	5.2^b^	47.3^a^	2.47	0.34	<0.001
C18:2 n-6	72.4	71.6	98.9^a^	18.3^b^	98.8^a^	4.65	0.36	<0.001
C18:3 n-3	38.2	38.3	40.9^a^	33.0^b^	40.9^a^	0.19	0.12	<0.001
Total	248	247	312^a^	119^b^	312^a^	10.48	0.45	<0.001
Milk FA, g/100 g ID FAME ^6^								
Individual FA								
C14:0	8.9	8.4	9.8^a^	6.2^b^	9.8^a^	1.16	0.10	<0.001
C16:0	26.7	25.9	27.3^a^	24.1^b^	27.4^a^	1.49	0.09	<0.001
C18:0	10.6	11	11.6^a^	11.4^a^	9.4^b^	1.14	0.31	<0.001
C18:1 cis-9	24.1	26.1	22.3^b^	30.2^a^	22.9^b^	2.55	0.002	<0.001
FA according to saturation								
Saturated FA	61.9	60.3	64.7^a^	55.6^c^	63.0^b^	2.95	0.04	<0.001
Monounsaturated FA	32.9	34.6	30.8^b^	38.8^a^	31.7^b^	2.6	0.01	<0.001
Polyunsaturated FA	5.2	5.1	4.5^b^	5.6^a^	5.4^a^	0.66	0.46	<0.001
FA according to origin								
De novo (C4:0 to C15:1)	22.1	20.8	23.4^a^	16.8^b^	24.1^a^	2.41	0.04	<0.001
Mixed origin (C16:0 + C16:1)	29.1	28.2	29.5^a^	26.7^b^	29.8^a^	1.48	0.09	<0.001
Mobilization (≥ C17:0)	48.8	51.0	47.2^b^	56.5^a^	46.1^b^	3.52	0.02	<0.001
C18:1 cis-9 to C15:0 ratio	16.6	19.2	15.5^b^	21.7^a^	16.5^b^	2.18	0.001	<0.001

^1^According to the clustering analysis based on pre-challenge cow traits and energy status.

^2^FP, feeding period.

^3^FA, fatty acid.

^4^ Residual standard deviation.

^5^ The interactions were not significant (*P =* 0.06–0.70).

^6^ Identified fatty acid methyl esters.

^a,b,c^ Different superscripts indicate differences among feeding periods (*P* < 0.05).

**Figure 5.**
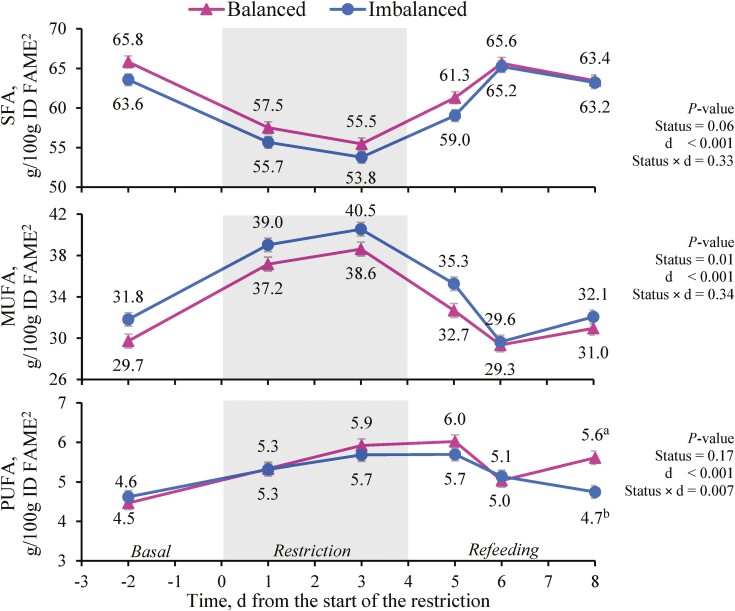
Effect of the status cluster^1^ and day (d) on beef cows’ milk concentrations of grouped fatty acids (FA) according to their saturation: saturated FA (SFA), monounsaturated FA (MUFA), and polyunsaturated FA (PUFA). The gray area represents the 4 d feed restriction at 55% of cows’ energy and metabolizable protein requirements. Vertical bars indicate the standard error. ^1^ according to the clustering analysis based on pre-challenge cow traits and energy status. ^2^ identified fatty acid methyl esters. ^a, b^ Within a day, different superscripts indicate differences between status clusters (*P* < 0.05).

When FA were analyzed according to their degree of saturation, both SFA and MUFA were affected by the status cluster (*P <* 0.05) and the feeding period (*P <* 0.001), and PUFA only by feeding period (*P <* 0.01; Table 6). The milk FA profile of Balanced cows had greater SFA and lesser MUFA contents than that Imbalanced cows, whereas PUFA contents were similar in both status clusters. During the restriction, SFA content lowered, while MUFA and PUFA rose (*P <* 0.001). During refeeding, SFA increased but did not reach the basal values, MUFA decreased to the basal values and PUFA remained unchanged. When analyzed by day, the SFA basal values had recovered by day 6 and after 2 d on the refeeding diet (Figure 5). For PUFA, a status cluster and day interaction was observed (*P =* 0.01, Figure 5) because Balanced cows had not regained the basal values by day 8, whereas Imbalanced cows had. Altogether, milk SFA contents correlated highly and positively with total diet FA intake and cow EB (*P <* 0.001; Figure 2), while negative correlations were observed between milk MUFA content and both parameters (*P <* 0.001). SFA ­negatively and MUFA positively correlated with NEFA plasma contents (*P <* 0.001).

**Figure 6.**
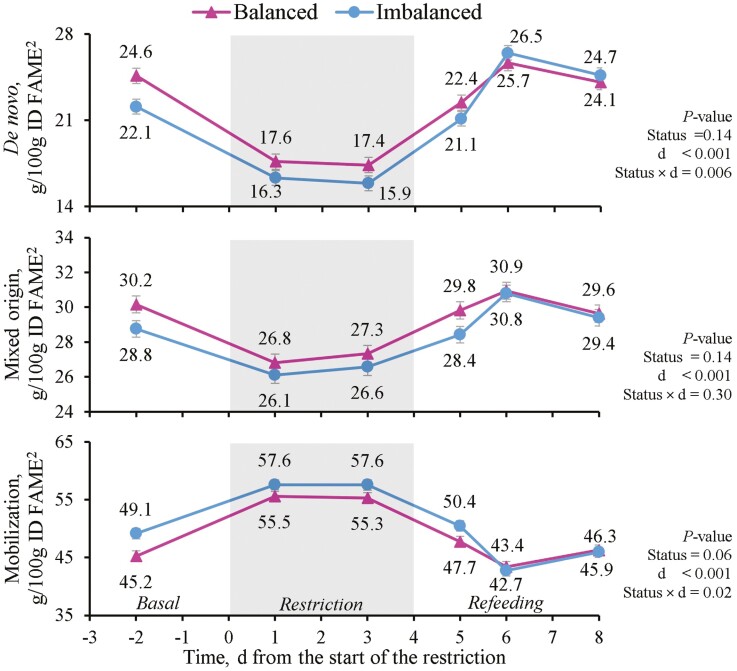
Effect of the status cluster^1^ and day (d) on beef cows’ milk concentrations of grouped fatty acids (FA) according to their origin: De novo FA (C4:0–C15:1), mixed origin FA (C16:0–C16:1), and mobilization FA (≥ C17:0). The gray area represents the 4 d feed restriction at 55% of cows’ energy and metabolizable protein requirements. Vertical bars indicate the standard error. ^1^ according to the clustering analysis based on pre-challenge cow traits and energy status. ^2^ identified fatty acid methyl esters.

Regarding the effect on the FA grouped according to their origin, the status cluster affected de novo (C4:0–C15:1) and mobilization FA (*P* < 0.05), and tended to affect mixed origin FA (C16:0–C16:1) (*P* = 0.09) with Balanced cows having greater de novo FA contents, slightly greater mixed origin FA and lesser mobilization FA than Imbalanced cows (Table 6). Feeding period affected the three FA groups (*P* < 0.001). De novo and mixed origin FA decreased, while mobilization FA increased during the restriction before returning to the basal values during refeeding. When analyzed by day, an immediate effect was noted on de novo FA during the restriction in both status clusters, with low and constant values on day 1 and 3 (Figure 6). They thereafter increased during refeeding to the basal values on day 5 in both status clusters, but continued to rise even beyond the basal values on day 6 and 8 in Imbalanced cows. Similarly, the daily values of mixed origin FA lowered immediately with the restriction and increased from the start of refeeding irrespectively of the status cluster (Figure 6). Mobilization FA of both Balanced and Imbalanced cows sharply rose on the first day of restriction (day 1), decreased with refeeding below the basal values on day 6 and returned to the baseline values on day 8 (Figure 6). Daily individual EB correlated highly and positively with milk contents of de novo and mixed origin FA (*P <* 0.001; Figure 2), but negatively with mobilization FA (*P <* 0.001). De novo and mobilization FA obtained correlations of a different sign with NEFA plasma concentrations (*P <* 0.001).

The C18:1 cis-9 to C15:0 ratio was affected by the status cluster (*P =* 0.001), with greater values in Imbalanced cows than in their Balanced counterparts, and also by the feeding period (*P <* 0.001) with an increment during the restriction and a return to the basal values during the refeeding period (Table 6). This ratio correlated negatively with EB (*P <* 0.001) and positively with plasma NEFA concentrations (*P <* 0.001), but not with the other plasma metabolites (Figure 2).

## Discussion

This study investigated the pattern of beef cows’ adaptive responses in different energy statuses to a short, but intense, feed restriction, and subsequent refeeding. Their pre-challenge performance and energy status were established by retrospective cow classification according to their previous BW, milk yield, and EB. We obtained two distinct status clusters: Imbalanced cows were heavier, tended to have greater milk yields and a more negative EB, whereas Balanced cows fed the same diets were lighter, had lesser milk yields and a neutral EB. When subjected to nutrient restriction, and despite wide between-cow variability, most of the parameters that describe cows’ performance, plasma metabolites, and milk composition were affected by time (feeding period or day). A less marked effect was observed for the status cluster (Balanced vs. Imbalanced cows).

### Cow performance

According to the experimental design, DMI (64%) and both energy (55%) and protein (53%) intake lowered during the restriction period, which resulted in lighter BW (−2%), lower milk yield (−17%), and less milk protein content (−3%) compared to the basal values. Milk fat content did not change, and milk urea content increased (+13%). The BW loss could be a consequence of the reduced DMI and the concomitant loss of gut fill, together with the mobilization of body reserves in response to the restriction (Gross et al., 2011a). This mobilization was probably larger for Imbalanced cows, which were heavier and had a lower EB throughout the study, which allowed them to cope with the metabolic challenge, but resulted in net BW loss at the end of the refeeding period.

The diminished milk yield during the restriction was associated with reduced energy supply, as observed in other studies. The −17% reduction herein observed for beef cows after a 4 d restriction at 55% of their requirements was similar to the −19% to −20% reduction after a 4–5 d restriction at 50–60% of previous intake for dairy cows (Carlson et al., 2006; Abdelatty et al., 2017). A greater (−30%) reduction was observed when dairy cows were restricted more intensely (48% of their requirements) for 4 d (Bjerre-Harpøth et al., 2012). In beef cattle, Charolais cows had −12% milk loss under a similar restriction condition, which was probably related to a less negative physiological imbalance (De La Torre et al., 2022). As observed here, all the aforementioned studies report a wide variation in cows’ individual adaptive ability to counterbalance the feed restriction, which Orquera-Arguero et al. (2022) associated to the cows milk yield potential and capacity of mobilization of fat reserves.

Despite the fact that the basal milk yield did not differ between status clusters, it was not only numerically greater in Imbalanced cows, as observed by De Koster et al. (2019) in two groups of cows clustered according to their metabolic profiles, but also recovered more quickly when refeeding started. According to Baumgard et al. (2017), milk yield would be a major driver of the different partition of nutrients toward milk production or fat reserves in cows and would, therefore, condition their response to feed restriction. The slower recovery observed in Balanced cows resulted in their EB being even better during refeeding than during the basal period because energy intake exceeded their requirements for a numerically lesser milk yield. When analyzed by day, the basal values had recovered in both status clusters by the end of the refeeding period. This finding agrees with other studies in beef (De La Torre et al., 2022) and dairy (Gross et al., 2011a; Bjerre-Harpøth et al., 2012) cows, which reflects the plasticity of the cow response to a short nutritional challenge.

Several studies report greater milk fat content associated with a negative EB and body fat mobilization (Agenäs et al., 2003; Kessel et al., 2008), whereas others report no difference between cows with different fat mobilization intensities (Schuh et al., 2019). In the present study, no changes were observed in response to a short feed restriction, which agrees with the results of Carlson et al. (2006), who worked with dairy cows under similar conditions, although they also found increased plasma indicators of lipolysis (NEFA and BHB). As pointed out by Schuh et al. (2019), the fact that milk fat did not mirror the increase in circulating NEFA could be explained by them being partly diverted to other tissues to be used as an energy substrate rather than to the mammary gland to be converted into milk FA. Milk fat content tended to be greater in Imbalanced cows, which agrees with the observations made by Stoop et al. (2009) when comparing cows with different EB, which could reflect a longer-term difference in the nutritional status of cows with different BW and milk yields fed at a flat rate since lactation onset.

The immediate milk protein content reduction during the restriction period observed in similar studies with dairy cows (Gross et al., 2011a; Billa et al., 2020) can be ascribed to reduced dietary energy and protein intake, which compromise both microbial protein synthesis and by-pass protein flux to the intestine. Similarly, Bjerre-Harpøth et al. (2012) confirmed that milk protein content lowered during the restriction and returned to the prerestriction content during refeeding regardless of the lactation stage. The rise in milk urea contents during feed restriction agrees with the observations made by Broderick (2003), who described that when dietary energy lowers, milk yields, and milk protein contents decrease, while milk urea increases, in response to the lower amino acid requirements for lesser milk secretion (Bittante, 2022).

### Blood metabolites

In the present experiment, the metabolites associated with energy metabolism and oxidative status were not affected by the status cluster, except for greater plasma urea concentration in Imbalanced cows. Glucose, NEFA, and urea immediately responded to diet changes, while a delayed response was noted for BHB and MDA. Plasma glucose concentration strongly depended on the current energy and protein intake at a given time, and also on diet composition. They were all similar for both status clusters and, thus, their glucose concentration did not differ. Plasma glucose did not change during the restriction, although it was expected to decrease as a consequence of lower feed and energy intake. This lack of response could be due to the lower gluconeogenesis associated with lower ruminal propionic acid production (Kessel et al., 2008) caused by the lower proportion of concentrate in the restriction diet. However, circulating glucose also depends on uptake by mammary glands for milk lactose production, as observed in other studies (Agenäs et al., 2003; Carlson et al., 2006). The increment that occurred in the refeeding phase agrees with the observations made by Bjerre-Harpøth et al. (2012), for whom glucose also peaked at the start of refeeding due to metabolic readjustment.

An increase in circulating NEFA concentration is an indicator of adipose tissue catabolism in response to a negative EB to supply FA, which can be converted into milk triglycerides in the mammary gland or oxidized in the liver as an energy substrate (Bell, 1995). In the current study, NEFA did not differ among cows in both status clusters, probably because the actual difference in EB between them was too narrow to elicit a response. However, they responded immediately to the large differences in energy intake among feeding periods, with which they correlated. A critical threshold of 0.57 mmol NEFA/L was set by Ospina et al. (2010) as an early postpartum indicator of increased risk of clinical ketosis in dairy cows, which was only just reached by Balanced cows on day 3 in our experiment.

Excessive NEFA mobilization can impair the liver’s metabolic capacity to completely oxidize them, which results in the production of ketone bodies, such as BHB, acetoacetate, and acetone (Jorjong et al., 2015; Mann et al., 2016). In our experiment, the tendency of a greater BHB concentration for Imbalanced than Balanced cows, plus the positive correlation between BHB and milk yield, suggest increased NEFA oxidation to provide energy substrates for milk production (Wathes et al., 2007). The BHB concentrations did not differ among feeding periods, as observed in dairy and beef cows at mid-lactation with a similar feed restriction period lasting 4 d (Carlson et al., 2006; Bjerre-Harpøth et al., 2012; De La Torre et al., 2022). These results imply that NEFA mobilization did not exceed the liver’s metabolizing capacity and provided sufficient energy supply for nutrient-restricted cows. However, a peak occurred at the end of the restriction phase, with a delayed response to energy intake compared to NEFA, as observed by Gross et al. (2011a) in dairy cows at mid-lactation. The extent of this delay can be influenced by the lactation stage and restriction duration (Carlson et al., 2006; Orquera-Arguero et al., 2022). Apparently, feed restriction length did not suffice here to have a prolonged effect on BHB. Plasma BHB can be used as an indirect marker of a negative EB in dairy cows, but has been shown to be a poor indicator in beef cattle (De La Torre et al., 2022; Orquera-Arguero et al., 2022), as observed here. Hyperketonemia, defined when BHB exceeds a critical threshold of 1.2 mmol/L, is associated with increased risk of disease, milk yield losses, and impaired reproductive performance in dairy cows (Jorjong et al., 2015). In our study, both NEFA and BHB concentrations were below the above-mentioned thresholds because our beef cows had a less severe negative EB due to their lower milk yields.

Lack of differences in these metabolites between status clusters was not expected. De Koster et al. (2019) observed that plasma glucose was greater and NEFA and BHB were lesser in balanced than in imbalanced dairy cows. Vossebeld et al. (2022) clustered cows according to their postpartum EB profile. They found that those with a more negative EB had greater plasma NEFA and BHB concentrations. However, differences in EB between the dairy cow groups in both studies, and associated with their different DMI, BW, and milk yield, were much larger than those herein recorded. Our similar results for both cow groups in different EB could be partly ascribed to wide individual variation in cows’ metabolic adaptive capacity, as pointed out by Kessel et al. (2008), or to the lower milk yield and associated metabolic load in beef cows.

Circulating urea in lactating ruminants originates from either dietary protein intake or the catabolism of body protein reserves when energy intake is restricted and the AA stored in skeletal muscle are mobilized (Bell, 1995). Given their similar protein intake, the greater plasma urea ­concentrations in Imbalanced cows indicate greater body protein turnover to support gluconeogenesis and to cope with their more negative EB. These differences observed in plasma were probably not large enough to be reflected in milk urea contents, despite them being significantly correlated, as observed by Kessler et al. (2020). The minor differences among days, which decreased at the end of the restriction and had risen by the end of the refeeding period, showed a delayed response to diet changes, which falls in line with Bjerre-Harpøth et al. (2012).

Oxidative stress occurs during periods of high metabolic demand, when the production of free oxidant radicals cannot be counteracted by the natural anti-oxidant system. Castillo et al. (2006) found increased lipid peroxidation only at very early postpartum, with wide individual variation. Bernabucci et al. (2005) reported that dairy cows with greater BCS loss, and greater BHB and NEFA concentrations, also had greater concentration of reactive oxygen metabolites, which agrees with Schuh et al. (2019), plus lesser concentrations of antioxidants. In our study, Imbalanced cows tended to have greater MDA concentrations, which mirrored the trend observed for BHB concentrations. This finding also reflects fat mobilization and oxidation, and is associated with hepatic stress. This positive correlation between MDA and BHB agrees with those observed by Li et al. (2016) in dairy cows, who also report a positive association with NEFA, but it was not observed in our experiment. This supports the lack of differences in oxidative status among feeding periods, where the increased NEFA and the decreased milk yield allowed cows to cope with metabolic stress without further lipid oxidation. In line with our results, Urh et al. (2019) found that diets that included different amounts of concentrate affected NEFA concentrations, but neither BHB nor the oxidative status of dairy cows, which they associated with relatively small differences in cows’ energy intake, as we observed here with a flat-rate feeding regime.

### Diet FA intake and milk FA content

The total FA intake decreased by −62% due to the restriction, whereas the extent of the decrease in individual FA intake varied, with a greater reduction (−81% to −89%) for those that were more abundant in the concentrate (C18:2 n-6 and C18:1 cis-9) than for those that were predominant in hay (C16:0 and C18:0). These differences in relative individual FA intake reflected both the reduction in DMI and the change in diet among periods. Diet composition affects the milk FA profile because short- and medium-chain milk FA derive from de novo synthesis from acetate and the transformations of butyrate that occur during the ruminal fermentation of carbohydrates (Bauman and Griinari, 2003), both of which increase when the forage proportion in diet increases. However, the milk FA profile does not exactly mirror the relative intake of the different FA because they can be modified by ruminal biohydrogenation and mammary lipogenic and Δ-9 desaturation pathways (Chilliard et al., 2007).

Research into the relation between energy intake and EB with the milk FA profile is extensive in dairy cows, but literature on milk FA composition of beef cows is scarce. To the best of our knowledge, this is the first study to report changes in beef cows’ milk FA contents in response to feed restriction. As in the case of milk yield and circulating metabolites, the response patterns of milk FA in beef cows follow the trends observed in dairy cows although the changes are of a lesser magnitude. Here, we observed that energy status had a marked effect in both the long (differences between status clusters, e.g. C14:0 and C16:0 tended to be greater and C18:1 cis-9 lesser in Balanced vs. Imbalanced cows) and short terms (differences among feeding periods, e.g., lowest C14:0 and C16:0 and highest C18:1 cis-9 during the restriction) on milk contents of major FA and different FA proportions according to both their degree of saturation and origin. When a negative EB induces body fat mobilization, the major FA in subcutaneous and abdominal depots (C16:0, C18:0, and C18:1 cis-9) are released to plasma, where they constitute a high proportion of circulating NEFA, and where C18:1 cis-9 is the most abundant FA in both dairy (Hostens et al., 2012) and beef (Lake et al., 2007) cows. These NEFA are taken up by the mammary gland and directly used for milk fat synthesis (Bauman and Griinari, 2003). Consequently, their relative proportions in milk fat should reflect this lipid mobilization in response to EB. Furthermore, when these long-chain FA are released into plasma, de novo synthesis of short-chain FA by the mammary gland is inhibited (Chilliard et al., 2007). Gross et al. (2011b) described how the milk FA profile responds quickly to dietary energy changes, with significant reductions in most FA of ≤C16:0 and increments of preformed FA of > C16:0 within 1 wk of feed restriction, and the basal values recover within 1 wk of refeeding. This pattern was confirmed in our experiment, even on the first day after diet change. As we noted, C14:0 milk contents were positively associated with EB, and increased with improved energy status with advancing dairy cows’ lactation (Craninx et al., 2008). On C16:0, literature offers conflicting results, which are explained by its mixed origin (Chilliard et al., 2000). C16:0 contents increased with either a negative EB (Stoop et al., 2009) or feed restriction (Abdelatty et al., 2017), but the decrease herein observed during the restriction period agrees with the patterns reported by Gross et al. (2011b) and Billa et al. (2020), which suggests that despite its mixed origin, here it reflects the reduced de novo FA synthesis. Regarding long-chain FA, milk C18:0 did not increase during the restriction, unlike previous reports (Gross et al., 2011b; Billa et al., 2020), but decreased with refeeding as a result of less fat mobilization, which agrees with the aforementioned studies. Finally, milk oleic acid contents (C18:1 cis-9) have been associated with a negative EB and high plasma NEFA concentrations (Stoop et al., 2009; Jorjong et al., 2014; Dórea et al., 2017), which agrees with our results. It has even been proposed as an early predictor of subclinical ketosis in dairy cows (Van Haelst et al., 2008), and as a better indicator of a negative EB than actual plasma NEFA and BHB concentrations (Churakov et al., 2021), which can vary diurnally depending on the time that elapses between feeding and blood sampling (Mäntysaari et al., 2019). This was confirmed herein by the stronger correlation of EB with milk C18:1 cis-9 contents than with these plasma metabolites. This relation also explains the greater milk contents of C18:1 cis-9 in Imbalanced cows, and the rise that occurred during the restriction period in association with a more negative EB in both cases.

According to their degree of saturation, the differences between status clusters and feeding periods followed the differences in major FA and in other less abundant ones. During the feed restriction, SFA decreased by −14% whereas MUFA and PUFA increased by +26% and +24%, respectively. This agrees with the results of Gross et al. (2011b) except for their stable PUFA concentrations, but contrasts with those of Stoop et al. (2009), who found greater proportions of SFA, mainly C16:0 and C18:0 from body fat, in those cows with a greater energy imbalance. The reduction in SFA during the restriction and the lesser concentration in SFA in Balanced cows in our study seemed to be driven by the predominant behavior of C16:0 as a de novo synthesized FA, and also by the minimal response of C18:0 to EB, as observed by Abdelatty et al. (2017). Regarding the origin of milk FA, Grummer (1991) suggests that almost all the C4:0 to C14:0, and about half the C16:0 in milk, are synthesized de novo in the mammary gland, whereas the rest of the C16:0 and all long-chain FA derive from mammary uptake of circulating triacylglycerol and NEFA. Unless diet composition significantly varies (Khiaosa-ard et al., 2020), the relative proportions of de novo synthesized and preformed FA mainly reflect changes in the EB (Gross et al., 2011b). Accordingly in our study, milk de novo FA content was significantly greater and that of mobilization FA was lesser in Balanced vs. Imbalanced cows. In dairy cows that underwent a 6 d 50% energy restriction, Billa et al. (2020) reported that milk contents C10:0 to C15:0 decreased by −37%, and those of C16:0 by −23%, while FA > C16:0 rose by almost +60%, and basal contents were recovered within a week of refeeding. Here with a similar but shorter feed restriction in beef cows, the relative changes were less intense, i.e., both de novo and mixed origin FA decreased (by −28% and −10%), while mobilization FA increased by +20%, and the basal values were also regained during the refeeding period in response to the improved EB. These changes are consistent with the strong correlations of the FA of different origins with EB and NEFA contents, as also described by Khiaosa-ard et al. (2020), who also found correlations with BHB contents that were not herein observed.

Several ratios between milk FA of different origins (mostly long-chain vs. short- and medium-chain FA or linear and branched FA) have been proposed as indicators related to cow diet or energy status (Craninx et al., 2008; Dórea et al., 2017). Of them, Jorjong et al. (2015) established that the C18:1 cis-9 to C15:0 ratio was the most discriminating factor for early hyperketonemia diagnosis (BHB ≥ 1.2 mmol/L), for which they proposed a threshold of between 34 and 45. Dórea et al. (2017) indicated that it could also be used to accurately predict plasma NEFA and that when this ratio exceeded 62, the cows would be at risk of developing metabolic disorders. In our experiment, the C18:1 cis-9 to C15:0 ratio differed between the status clusters and feeding periods by following the differences observed in EB and plasma NEFA contents, with which it correlated, and could therefore be used as a biomarker of the energy status of cows. However, our values were far from the above-mentioned thresholds described for dairy cows.

## Conclusions

A short-term feed restriction and refeeding induced a transient negative EB in beef cows, to which they responded with lower milk yield and changes in plasma metabolites and milk composition, which are associated with the mobilization of body reserves. Despite some of these traits differing between Balanced and Imbalanced cows, with different BW, milk yields and EB before the challenge, they responded similarly to dietary changes by showing a consistent pattern across several individual nutritional statuses. The milk FA profile, which has been rarely studied in beef cows for practical purposes, also differed between Balanced and Imbalanced cows. In particular, the milk C18:1 cis-9 to C15:0 ratio proved to be an accurate indicator of metabolic status, which supports its use in experimental models.

## Abbreviations

BCS: body condition score
BHB: β- hydroxybutyrate
BW: body weight
DIM: days in milk
DM: dry matter
DMI: dry matter intake
EB: energy balance
FA: fatty acids
FAME: fatty acid methyl esters
MDA: malondialdehyde
MUFA: monounsaturated fatty acids
NE: net energy
NEFA: nonesterified fatty acids
PUFA: polyunsaturated fatty acids
SFA: saturated fatty acids
